# How does age discrimination limit the mental health benefits of active aging?

**DOI:** 10.1093/geroni/igaf122.2344

**Published:** 2025-12-31

**Authors:** YoungBin Koh, Yeonjung Lee

**Affiliations:** Chung-Ang University, Seoul, Republic of Korea; Chung-Ang University, Seoul, Republic of Korea

## Abstract

Activity theory suggests that participating in social activity or having a job improves overall physical and mental health in older people. Meanwhile, perceived age discrimination is a substantial stressor because they can adversely affect mental health. It is known that specific major events of discrimination are often tied to life-course experiences that are less likely to occur in later life. However, as the benefits of active aging have been emphasized, more older adults are engaged in employment and social activity, which they might be exposed to the potential stressor of experiencing discrimination due to their age. Since the Senior Employment and Social Activity Program (SESAP) was introduced in Korea, many studies have evaluated the effects of programs in various ways. However, not much attention has been paid to whether age discrimination maybe get in the way of benefitting from participation in SESAP. The purpose of this study is to examine the moderating effect of perceived age discrimination on the relationship between participation in SESAP and depression. Data from the Senior Citizen Survey in 2020 was (N = 9,201). Results show that the association between participation in SESAP and depression is significant and negative, but only at a low level of age discrimination. Essentially, age discrimination negates the mental health benefits of participating in work and social activity in later life. Findings have implications for policy and practice such as strengthening the laws to prevent age discrimination and expanding education programs to raise awareness of the consequences of age discrimination.

